# Cicada Wing‐Inspired Skeleton‐GO Networks in Melamine Foams for Multifunctional High‐Performance Composites

**DOI:** 10.1002/advs.76720

**Published:** 2026-07-24

**Authors:** Jigang Feng, Anhao Li, Babak Safaei, Yun Kong, Yizhen Miao, Zhaoye Qin, Fulei Chu

**Affiliations:** ^1^ State Key Laboratory of Tribology Department of Mechanical Engineering Tsinghua University Beijing China; ^2^ Department of Mechanical Engineering Eastern Mediterranean University Famagusta Türkiye; ^3^ Department of Machining Assembly and Engineering Metrology Faculty of Mechanical Engineering VSB‐Technical University of Ostrava Ostrava Czech Republic; ^4^ School of Mechanical Engineering Beijing Institute of Technology Beijing China

**Keywords:** damping, graphene, mechanical, melamine foam, sound absorption, water proof

## Abstract

Foams with high damping capacity have high potential in noise reduction, vibration mitigation, and energy dissipation applications. However, integration of superior damping, strong mechanical properties, and effective sound absorption, into lightweight foams is still a serious challenge. Inspired by cicada wing structure, a new approach was developed to improve foam characteristics by establishing an in situ skeleton‐GO network within the foam. This bioinspired network architecture partially spans the open regions defined by adjacent foam skeletons, rather than coating the skeleton or fully filling pores. This method remarkably improved the properties of the foam such that vibration attenuation time was decreased by about 83.3%, damping property was increased by about 179.3%, sound absorption was effectively enhanced, and compressive modulus was increased by about 924%, allowing the foam to tolerate 1000 times its own weight without noticeable deformation. The originally non‐waterproof melamine foam becomes hydrophobic after modification, presenting contact angles of up to 114.2°. This research provided a new and versatile approach to improve synergistic multifunction of foam materials.

## Graphical abstract

Inspired by the intricate microstructure of cicada wings, researchers developed a novel in situ skeleton‐graphene membrane architecture within lightweight foams. This bioinspired design dramatically enhances multifunctional performance: vibration attenuation time decreases by ∼83.3%, damping capacity increases by ∼179.3%, and compressive modulus rises by ∼924%. Additionally, the originally hydrophilic melamine foam becomes hydrophobic with a contact angle of 114.2°.

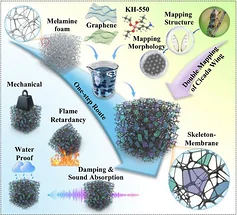

## 1 Introduction

Foams are being extensively used in sports, aerospace, biomedical, and consumer products [1, 2, 3, 4, 5, 6]. Particularly, because of their unique energy dissipation mechanisms and porous architecture, foams present significant advantages in sound absorption and vibration attenuation applications [7, 8, 9]. A great amount of research has been performed to improve the acoustic performance and vibration damping of foams [10, 11, 12, 13, 14]. However, conventional reinforcement approaches face notable limitations due to the fact that such enhancements should not compromise the inherent low‐density advantage of foams [12, 15, 16].

Recently, graphene has been commonly used to enhance the damping properties of foams as a 2D material with extraordinarily high specific surface area. Graphene‐reinforced foams have been prepared using a variety of methods, such as dip‐coating [13, 14, 17], blending [18, 19], and chemical vapor deposition [10, 11]. Constructing graphene networks on foam skeletons is a more representative method. In particular, Yu et al. [20, 21, 22] effectively enhanced the sound absorption and electromagnetic shielding properties of foam by constructing graphene networks on a porous framework. Dai et al. [23] constructed ultrathin graphene membranes in polymer foam, effectively enhance the sound absorption performance. Moreover, Pang et al. [24] constructed highly ordered graphene foams, effectively reducing junction thermal resistance.

Despite the fact that recent research works have partially solved the issue of excessive increase of weight in reinforced foams, the intrinsic superior features of foams have further potential for synergistic and simultaneous enhancements. Most previous studies have mainly focused on the targeted improvement of a single feature or, at most, two features including damping, sound absorption or mechanical performance. Therefore, there exists a strong demand for a simple yet unconventional strategy which can comprehensively unlock multiple functional properties of foams.

A Chinese proverb says, “as thin as a cicada's wing,” which vividly describes the extremely thin and light nature of cicada wings. Due to their unique structural properties, cicada wings present remarkable chemical, physical, and biological characteristics [25, 26, 27]. Research has shown that a regularly arranged nanopillar structure covers cicada wing surface at microscale, endowing them with strong hydrophobicity [28, 29]. Cicada wings are flexible and thin, allowing them to resist elastic recovery and cyclic deformation during flight while simultaneously enabling high‐frequency vibrations to generate sound [30]. These structural features have great potential for the multifunctional enhancement of foams. Structurally, cicada wings contain a supporting framework and a membrane layer consisting of epicuticle and cuticle [31]. From a structural analogy perspective, this study draws inspiration from the framework‐supported membrane structure of a cicada's wing. By constructing network regions within the open space enclosed by adjacent foam skeletons, the foam can synergistically enhance multiple functional properties while maintaining a lightweight structure.

Inspired by cicada wing structure, this research proposed a one‐step approach for enhancing foam performance. Using a single processing route, the sound absorption, damping, and mechanical properties of melamine foam were improved simultaneously, while introducing waterproof functionality. As presented in Figure 1a, based on the natural skeleton‐membrane architecture of cicada wings and extending it into three dimensions, a hybrid foam composite was fabricated by assembling membrane‐like graphene oxide (GO) network, with silane coupling agent interfacial bridges. Unlike conventional coating approaches, the GO is not to uniformly cover the skeleton surface or completely fill the cores of foam; instead, they formed locally connected and pore‐spanning assemblies networks supported by the foam skeleton.

**FIGURE 1:**
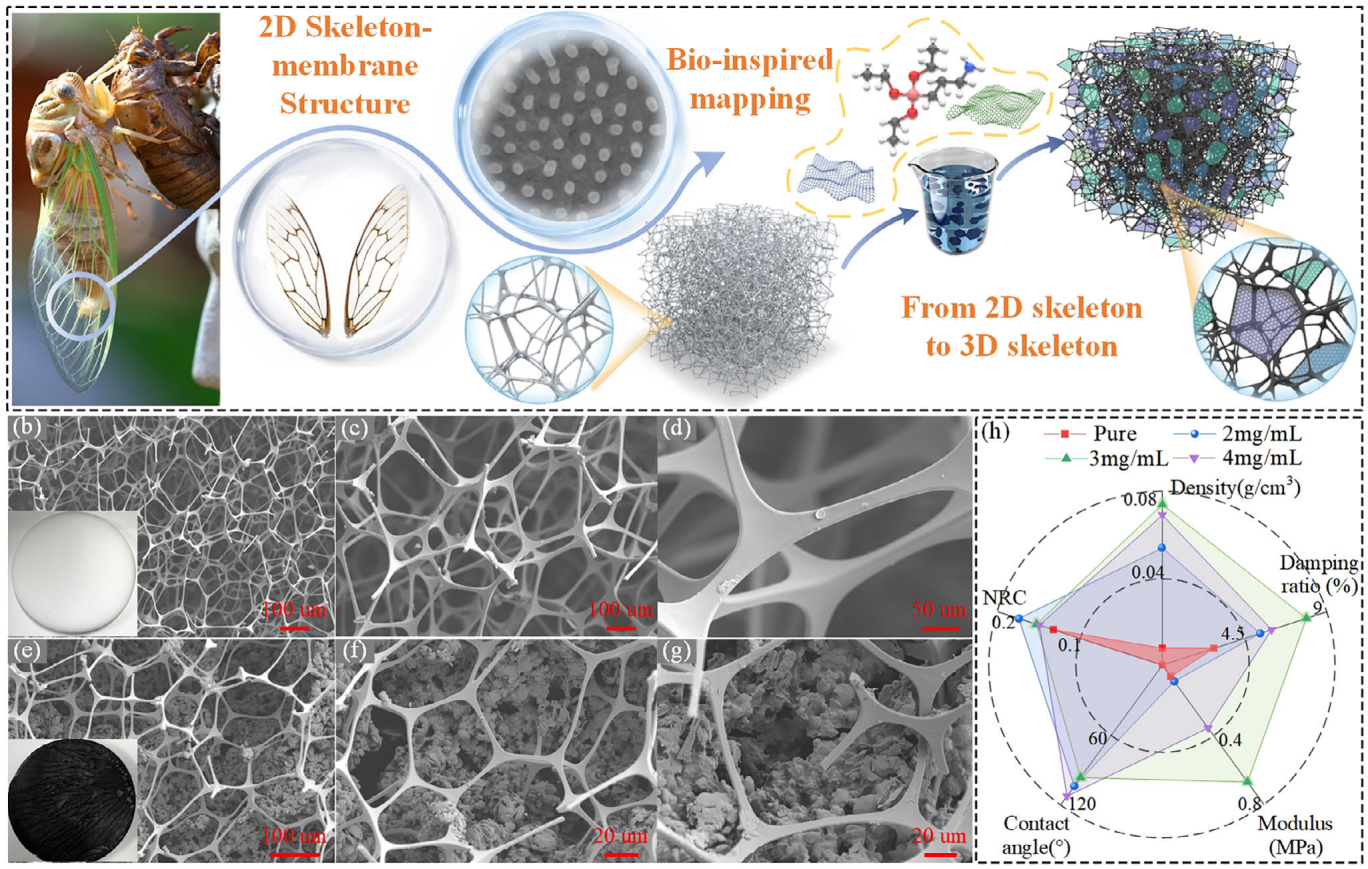
Schematic illustration of the skeleton‐GO networks strategy for enhancing the properties of foam structures. a) Schematic diagram of the cicada‐wing‐inspired skeleton‐mapping enhancement strategy. b‐d) Scanning electron microscopy (SEM) images of pristine melamine foam. e‐g) SEM images of melamine foam incorporating skeleton‐GO network. h) Comparison of multiple properties of melamine foam before and after enhancement.

This unconventional reinforcement approach effectively activated and synergistically regulated multiple functional features of melamine foam. As presented in Figure 1h, damping properties were enhanced by about 179%, accounting for vibration attenuation time reduction of about 83%. Regarding mechanical features, compressive modulus exhibited a maximum increase of 923%. Furthermore, it was noteworthy that this modification approach simultaneously endowed melamine foam with waterproof characteristics, significantly broadening its potential application scope.

## 2 Results and Discussion

### 2.1 Structural Characterizations

As shown in Figure 1b–d, pristine melamine foam exhibited a relatively dense and continuous skeleton structure. After modification, GO was supported by the foam skeleton and “rested” on the spatial planes formed by different skeleton units. It could be clearly observed that GO aggregation occurred and the GO networks formed among the skeletons were not perfectly flat. Furthermore, as illustrated in Figure S1a–c, excellent interfacial adhesion was observed both among adjacent GO sheets and between the GO networks and the melamine foam skeleton. In addition, as shown in Figure S1d–f, locally smooth GO network regions were observed. These smooth structures were mainly attributed to the self‐condensation of KH‐550, which formed siloxane networks that spread among foam skeletons [32]. As shown in Figure S2, a higher magnification local image, further demonstrates that this membrane‐like structure has excellent integrity, with obvious adhesion between the GO sheets, indicating that it forms a locally connected sheet‐network structure with a certain degree of structural stability. However, such siloxane‐derived films accounted for only a small fraction and did not dominate the overall structure of the composite. These observations clearly indicated that GO formed a membrane‐like structure within the melamine foam. Rather than merely coating the foam skeleton or completely filling the foam pores, the GO sheet networks partially bridged the open regions defined by adjacent skeletons. Nevertheless, it should be emphasized that a considerable number of skeleton‐formed planes inside the foam remained uncovered by GO networks. Figure S3 presents the surface morphologies of the foams fabricated with different GO concentrations. In low‐magnification SEM images, it could be observed that the coverage fraction of inter‐skeleton regions bridged by GO networks increased with GO concentration. When GO concentration reached 4 mg/mL, only a small number of pore regions remained unenclosed.

Figure S4 shows the results of the Thermogravimetric Analysis (TGA), it can be seen that in the temperature range of 380°C–400°C, pure melamine foam enters the main thermal oxidation weight loss stage, and its mass decreases rapidly, indicating that the foam skeleton has undergone violent thermal oxidation decomposition [33, 34]. In contrast, the GO network‐integrated sample showed a significantly slower rate of mass decrease in the same temperature range. Based on the characteristics of TGA in an air atmosphere, we believe that the mass loss mainly comes from two parts: first, the release of volatile products such as moisture and small molecule pyrolysis products; and second, further oxidation and ablation of carbonaceous residues. Below 200°C, for GO‐network‐integrated melamine foam, GO and silane coupling agent first undergo weight loss, deoxidation, and structural rearrangement, potentially forming interfacial residual structures on the foam skeleton surface; silane coupling agents may form Si‐O‐Si residues after heat treatment [35]. In the 200°C–350°C range, these interfacial structures may regulate the thermal oxidative decomposition pathway of melamine foam, promoting the condensation of some decomposition intermediates into residual substances, rather than complete conversion into volatile products. In the 350°C–500°C range, pure melamine foam undergoes faster thermal oxidative ablation, while for GO‐network‐integrated melamine foam, due to the presence of a more stable residual skeleton induced by GO and coupling agents, exhibit a slower mass loss rate and higher mass retention [36].

Figure S5 shows the Fourier transform infrared (FTIR) spectra of various specimens and GO. A broad absorption band of approximately 3400 cm^−1^ was observed in the FTIR spectrum of GO, corresponding to the O─H stretching vibration; an absorption band of approximately 1720 cm^−1^ was attributed to the C═O stretching vibration; and an absorption of approximately 1620 cm^−1^ could be attributed to sp2 carbon skeleton vibrations [37]. In addition, characteristic absorptions related to C─O─C were also present in the 1220–1050 cm^−1^ region, which further supports the indication that the GO surface contains abundant oxygen‐containing functional groups, which may include epoxy structures that can react with silane coupling agents [38]. Moreover, compared to the pure sample, the samples with GO networks exhibit enhanced absorption bands in the broad 3200–3500 cm^−1^ region, indicating enhanced polar group‐related vibrations at the interface. Simultaneously, the modified sample shows a more pronounced absorption band in the approximately 1100–1120 cm^−1^ region, which gradually increases with increasing GO content, particularly noticeable in the 4 mg/mL sample. This absorption characteristic is consistent with the formation of Si‐O‐Si structures [39], indicating that the silane component introduced by KH‐550 participates in the construction of the networks [40]. On the other hand, compared to the original GO, the samples with GO networks also show changes in the absorption characteristics in the 1220‐1050 cm^−1^ region related to GO oxygen‐containing functional groups, indicating that the oxygen‐containing groups on the GO surface underwent chemical environment reconstruction during the fabrication process.

The chemical structures of the pure and 4 mg/mL samples, as well as GO were analyzed using X‐ray photoelectron spectroscopy (XPS). Figure S6a compares the full XPS spectra of GO, pure, and 4 mg/mL. It can be seen that GO and pure melamine foam do not contain Si, while Si was detected in the 4 mg/mL. At the same time, the N1s in the 4 mg/mL sample also changed compared to the pure melamine foam. Furthermore, as shown in Figure S6b, in addition to C─C at 284.6 eV, C─O─C and C═O were also detected in GO at 286.1 and 288.9 eV, respectively [38]. This indicates that the oxygen‐containing functional groups on the GO surface can react further with the silane coupling agent to promote the formation of GO networks from single GO sheet. As shown in Figure S7, the high‐resolution C1s spectrum of the Pure can be decomposed into three components at 284.6, 285.9, and 287.8 eV, corresponding to C─C, C─N, and N─C═N carbon environments, respectively. In contrast, the C1s spectrum of the 4 mg/mL sample shows peak positions at 284.6, 285.5, and 286.7 eV, with 285.5 eV belonging to the C─N related carbon environment and 286.7 eV corresponding to the C─O related component. Compared to the original GO C1s spectrum, the high‐binding‐energy carbon oxide component at 288.9 eV is no longer retained in its original form in the composite sample, indicating that the oxygen‐containing functional groups on the GO surface were consumed during the fabrication process. Meanwhile, the N1s spectrum changed from 398.9 eV in the Pure sample to 399.3 eV in the 4 mg/mL sample, indicating that the nitrogen‐containing chemical environment changed after modification. In summary, the surface chemical state of the 4 mg/mL sample is not a simple superposition of the original GO and melamine foam, but rather a new interfacial chemical environment formed during the fabrication process. This result is consistent with the preparation mechanism in which KH‐550 participates in interfacial construction and GO oxygen‐containing functional groups participate in interfacial interactions [40]. Si2p appeared in the sample of 4 mg/mL, with a peak position of approximately 102 eV [39], as shown in Figure S8, which further supports the evidence that the silane coupling agent is involved in the adhesion process of GO membrane.

### 2.2 Exceptional Mechanical Properties of GO‐Network‐Integrated Melamine Foam

After the incorporation of GO networks, the mechanical properties of melamine foam was significantly enhanced. As shown in Figure 2a, both the 3 mg/mL and pure samples had the same initial thickness of 10 mm. When a load of approximately 10 kg was placed on top of the samples, the pure sample underwent pronounced deformation, with its thickness decreasing from 10 to about 6 mm, as illustrated in Figure 2b. In contrast, as shown in Figure 2c,d, the 3 mg/mL sample exhibited no obvious deformation under the same load (approximately 1000 times its own weight). This comparison demonstrated substantial enhancement in the load‐bearing capacity and mechanical stability of the melamine foam after GO networks introduction.

**FIGURE 2:**
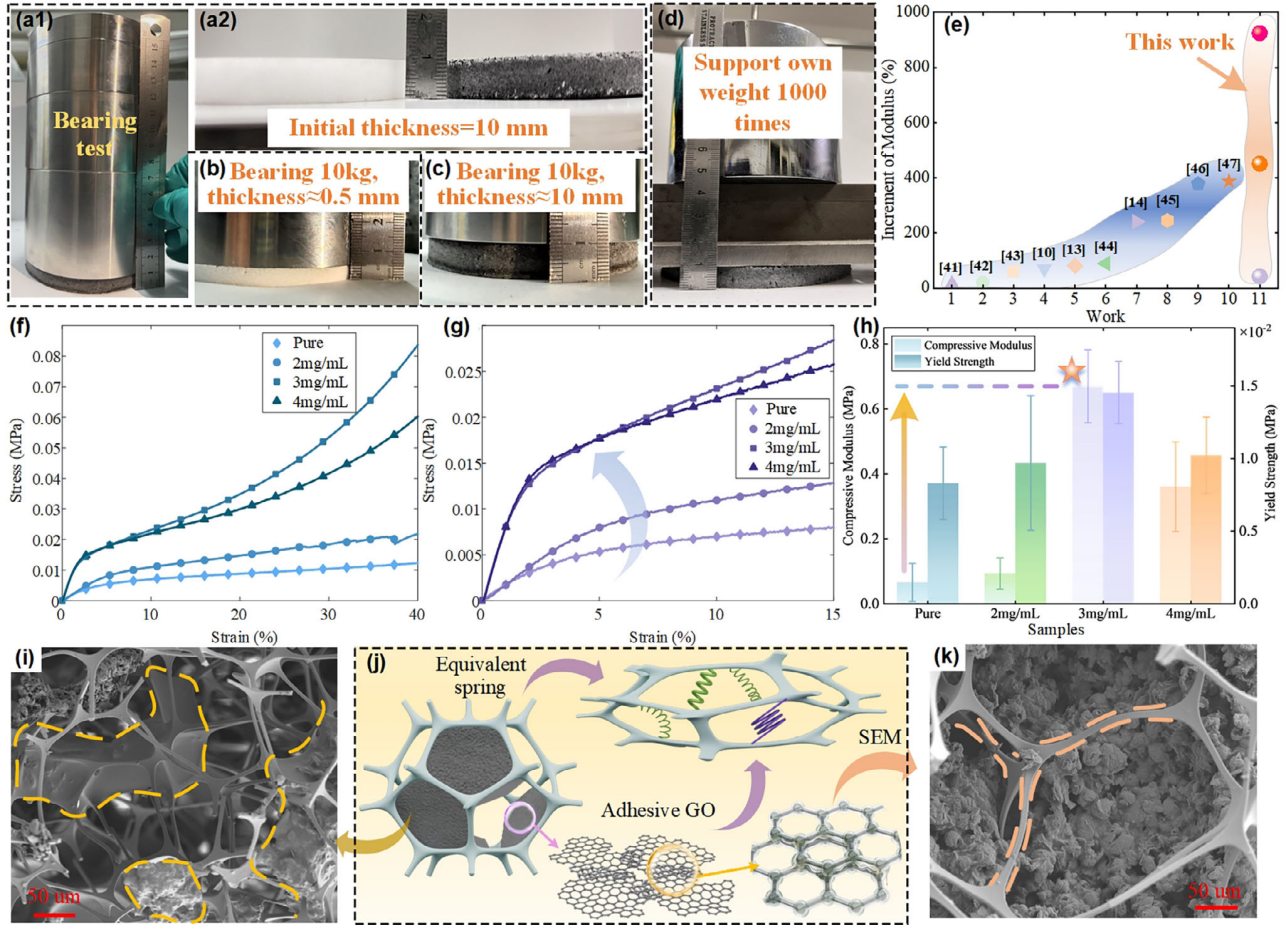
Mechanical properties enhancement of foam structures. a) Optical microscopy images showing the initial thickness of samples with and without GO networks. b) and c) Optical microscopy images of samples: b) without and c) with GO networks under a load of 10 kg. d) Load‐bearing optical image of the GO network‐integrated sample under higher loading. e) Comparison of the enhancement in compressive modulus achieved in this work with previously reported studies. f) Quasi‐static compressive stress‐strain curves. g) Enlarged view of the stress‐strain curves in the low‐strain region. h) Comparison of compressive modulus and yield strength (Data in panel h are presented as mean ± SD, with n = 3 independent specimens for each group. Error bars represent SD). i‐k) Schematic illustration of the mechanisms responsible for the mechanical properties’ enhancement induced by the GO networks.

The stress‐strain curves obtained from compression tests are shown in Figure 2f and the magnified views of the stress‐strain curves within 0–15% strain range are illustrated in Figure 2g. Compared with the pure sample, GO network‐integrated melamine foam exhibited greater slopes in the initial linear deformation region, along with increased yielding points. These results indicated that GO networks introduction significantly enhanced both the yield strength and compressive modulus of the melamine foam. Figure 2h shows the mechanical characteristics obtained from stress‐strain curves. The compressive modulus of GO network‐integrated melamine was increased from 65.5 MPa for pure to a maximum value of 669.9 MPa, corresponding to 924% enhancement. Yield strength was also significantly enhanced, increasing from 8.3 MPa for pure to 14.5 MPa at 3 mg/mL GO concentration, presenting an enhancement of about 74.7%. These findings demonstrated that GO networks incorporation effectively improved foam structure load‐bearing capability under compressive loading. Meanwhile, an increase in compressive modulus indicates higher equivalent stiffness of the foam. When foam is used to support a structure, the system corresponds to a higher natural frequency, which helps to optimize the dynamic response of the structure.

Comparison of the mechanical modulus enhancement achieved in this work with previous works are shown in Figure 2e [10, 13, 14, 41, 42, 43, 44, 45, 46, 47]. It was seen that the enhancement strategy developed in this research exhibited a clear advantage in the improvement of foam structure mechanical properties, especially at higher concentrations of GO. Furthermore, storage modulus variation as a function of frequency for different samples is illustrated in Figure S9. Storage modulus trends among samples with various concentrations of GO were consistent with those obtained from compression tests. Especially, the storage moduli of pure and 2 mg/mL samples remained almost constant over the tested range of frequency, while those of 3 and 4 mg/mL samples were increased with frequency. This could be due to the formation of more stable GO networks at higher concentrations of GO, facilitating more efficient stress transfer under high‐frequency loading, increasing storage modulus.

As presented in Figure 2i (3 mg/mL) and Figure 2k (4 mg/mL), both the siloxane networks formed by KH‐550 self‐condensation and the GO networks were observed to form continuous covering structures on the melamine foam skeleton and to maintain intimate interfacial contact with the skeleton. When the foam structure underwent deformation under external loading, these pore‐spanning GO networks could act as additional stiffness elements connected in parallel with the original skeleton, as schematically illustrated in Figure 2j, thereby effectively restraining the deformation of the skeletons. On the one hand, GO and siloxane network possessed relatively high intrinsic mechanical properties and could directly bear a portion of the applied load. On the other hand, locally connected GO assemblies facilitated stress transfer between adjacent skeletons, playing a role analogous to stress bridging. The synergistic contribution of load bearing and stress bridging resulted in the pronounced enhancement of the overall mechanical properties of the foam structure. To more clearly reveal the role of GO networks in enhancing the mechanical properties of melamine foam, nanoindentation of GO networks was conducted. Specific test details are listed in SI. As shown in Figure S11, For the 4 mg/mL sample, the GO networks maintained good integrity even at a compression depth of 10 µm. As shown in Figure S12, when the compression distance reaches 10 µm, the resistance load generated by the GO networks reach 0.6 N. It is the accumulation of this local resistance load that makes the melamine foam exhibit improved mechanical properties as a whole.

### 2.3 Rapid Vibration Attenuation Capability

Figure 3a illustrates the schematic diagram of the vibration testing setup used in this work. During the test, the sample acted as a supporting structure, with its bottom fixed to the testing platform. A 5 kg mass block was placed on top of the sample and an acceleration sensor was attached to the mass block to record the vibration response. Excitation signals were generated by a hammer and response signals were measured using the acceleration sensor. The first‐order damping ratio of the system was subsequently calculated using half‐power bandwidth method. From a mechanical perspective, the system could be equivalently modeled as a classical spring–mass system. By measuring the vibration attenuation behavior of the mass–foam assembly during free vibration, the damping ratio of the foam structure could be quantitatively determined. Comparative vibration attenuation curves of different samples are presented in Figure 3b–d. Specifically, under identical initial vibration amplitudes, the 2 mg/mL sample exhibited a higher attenuation speed than the pure sample, allowing it to more rapidly reach a stable state. The attenuation speed of the 4 mg/mL sample was further increased compared with that of the 2 mg/mL sample, while the 3 mg/mL sample showed an even faster attenuation speed than the 4 mg/mL sample. Among all samples, the 3 mg/mL sample demonstrated the most pronounced vibration attenuation behavior, as shown in Figure 3e, requiring only 0.19 s for transition from the vibrating state to a stable state. In contrast, this took 1.21 s for the pure sample, indicating that vibration duration was decreased by approximately five‐sixths. These vibration decay results clearly demonstrated the superior damping enhancement effect of the introduced GO networks on melamine foam. To more intuitively perceive the damping enhancement effect, the vibration attenuation signals of the pure and 3 mg/mL samples were processed and converted into sound files, as shown in sound SI and sound SII in the Supporting information, respectively.

**FIGURE 3:**
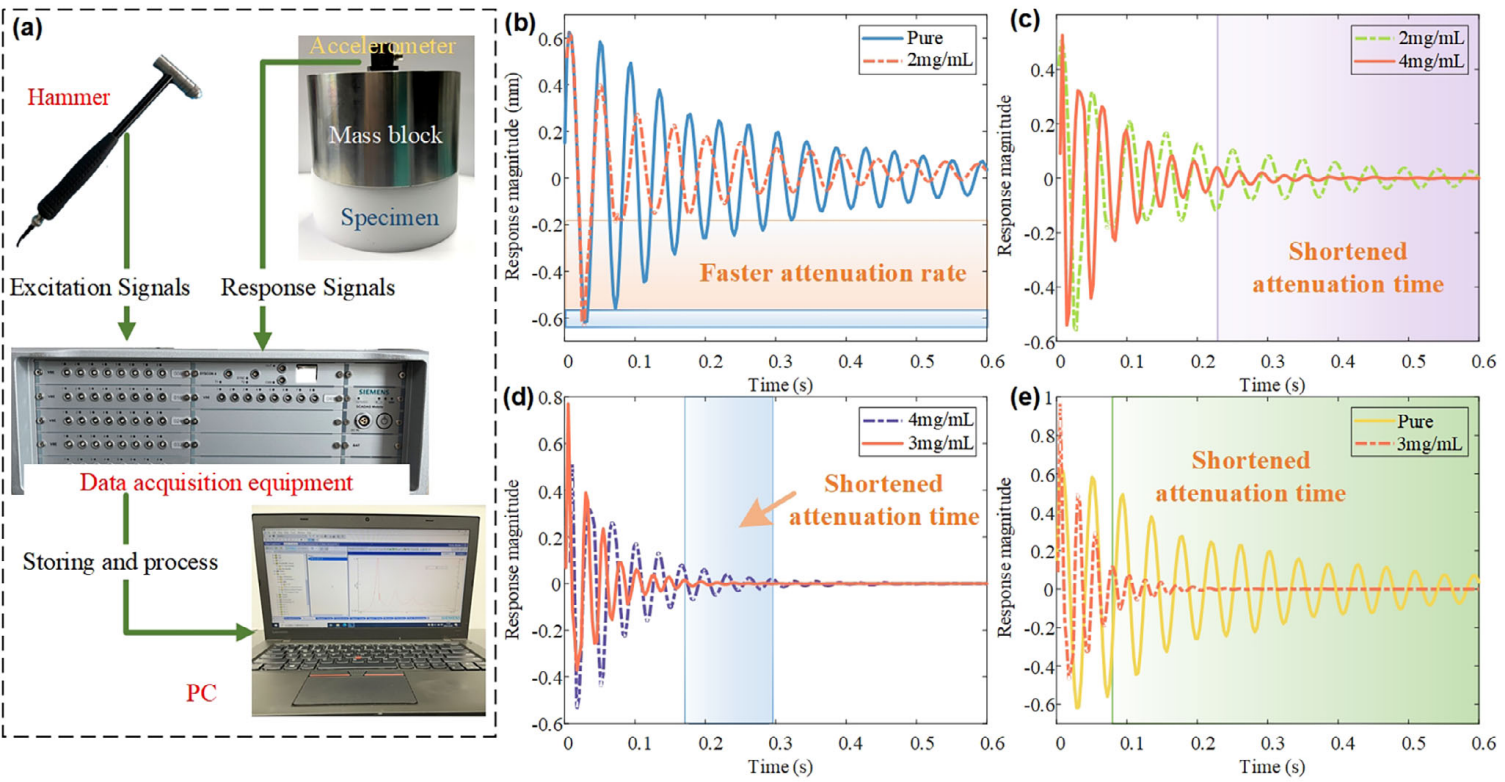
Damping properties characterization and vibration attenuation behavior of the foam structures. a) Schematic illustration of the testing setup of damping properties. Comparison of vibration attenuation curves between b) pure and 2 mg/mL samples; c) 2 and 4 mg/mL samples; d) 4 and 3 mg/mL samples; and e) pure and 3 mg/mL samples.

The first‐order damping ratios of the system were calculated based on vibration attenuation curves, and the results are presented in Figure 4a. The first‐order damping ratios of the pure, 2, 3, and 4 mg/mL samples were 2.822%, 5.354%, 7.881%, and 5.98%, respectively. Notably, the introduction of GO network increased the damping ratio of the melamine foam by up to 179.3%. In addition, the measured natural frequencies of the 3 and 4 mg/mL samples were 44.171 and 32.47 Hz, respectively, which were both significantly higher than that of the pure sample (23.92 Hz). As the vibration system can be reasonably approximated as a single‐degree‐of‐freedom spring‐mass system, its natural frequency can be expressed as $\omega^{2}=\frac{k}{m}$, where *k* and *m* denote the equivalent stiffness and mass of the system, respectively. Therefore, the increase of natural frequency also provided indirect evidence for the enhancement of the mechanical performance of the foam structure. Complementary to this, vibration attenuation behavior is determined by the combined dynamic effects of stiffness and damping properties; a higher compressive modulus also promotes a faster vibration attenuation speed for the foam. Therefore, the excellent vibration attenuation performance of the 3 mg/mL sample is attributed to the combined effect of structural stiffness and internal damping. To further demonstrate the advantage of the proposed approach in improving the damping properties of the foam structure, a comparison between the findings of this work and previously reported studies is presented in Figure 4c, which clearly demonstrates the superior enhancement achieved in this work [7, 8, 9, 11, 13, 14, 48, 49, 50, 51].

**FIGURE 4:**
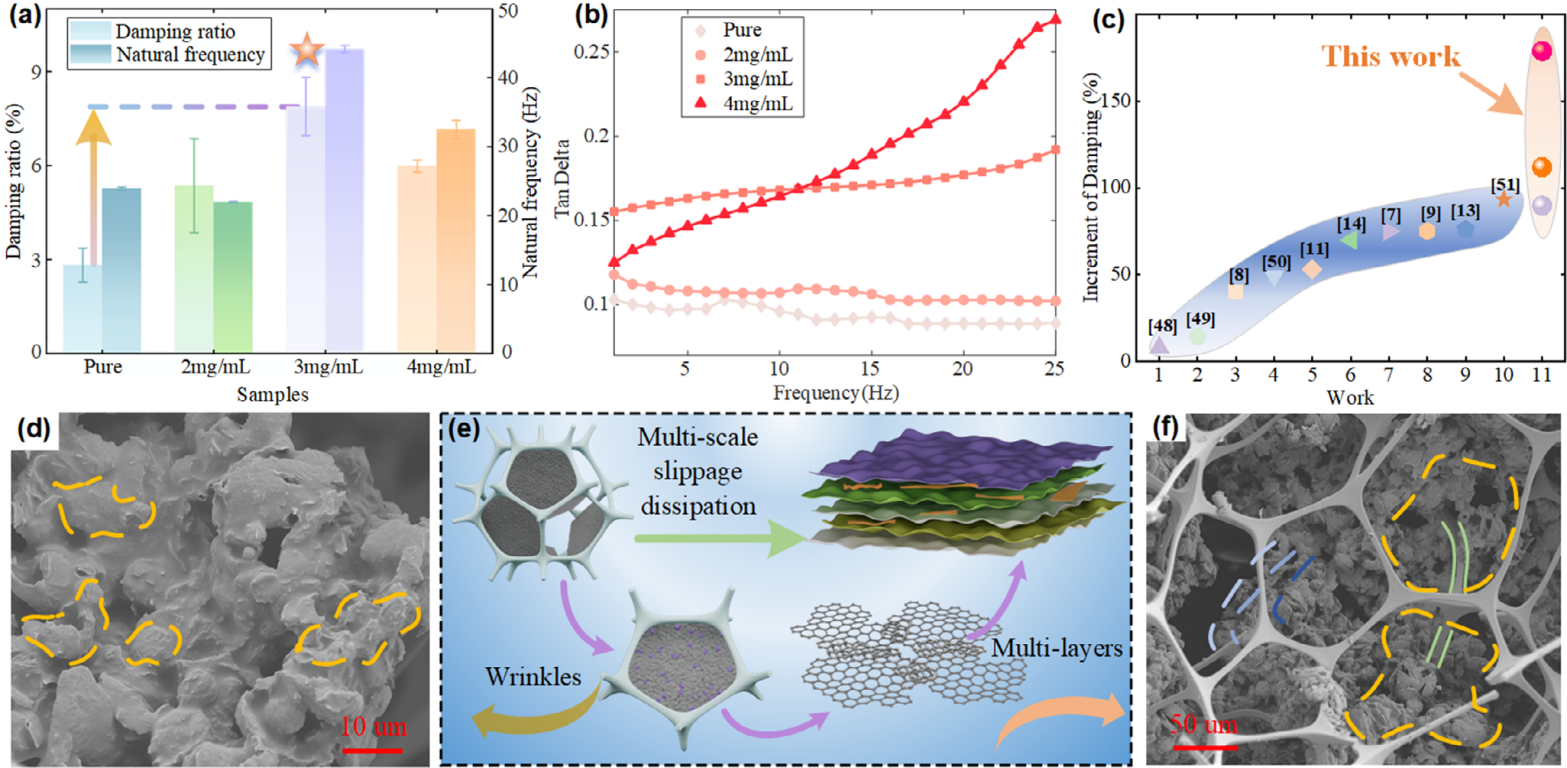
Damping properties and enhancement mechanisms of the foam structures. a) First‐order damping ratio and natural frequency (Data are presented as mean ± SD, with n = 3 independent specimens for each group. Error bars represent SD, The star symbols highlight the maximum values). b) Frequency‐dependent loss factor. c) Comparison of damping enhancement achieved in this work and previously reported studies. d), e), and f) Schematic illustrations of the mechanisms underlying the damping enhancement induced by GO networks.

Figure 4b presents the variation of the loss factor as a function of frequency for different samples. For the pure and 2 mg/mL samples, loss factor remained nearly constant over the tested frequency range. In contrast, for the 3 and 4 mg/mL samples, loss factor exhibited an approximately linear increase with the increase of frequency and higher GO concentrations led to larger slopes of the curve. When the foam structure was subjected to vibrational loading, microscale relative motions occurred within the foam skeleton and energy was dissipated primarily through the intrinsic viscoelasticity of the material. For the pure and 2 mg/mL samples, energy dissipation mainly originated from the foam skeleton itself. By increasing GO concentration, a greater amount of GO networks participated in the vibration‐induced energy dissipation process. Specifically, at higher concentrations, the foam skeleton interpenetrated and was embedded between GO networks. During microscale skeleton motions, GO networks were driven to deform and move cooperatively to introduce additional viscoelastic contributions from GO networks and enhance energy dissipation. In addition, as presented by the gradient blue dashed lines in Figure 4f (3 mg/mL sample), GO networks had a finite thickness, implying that mutual slippage among overlapped GO within the networks could take place under dynamic load, giving rise to extra energy dissipation. To verify this enhanced energy dissipation mechanism, we conducted in situ dynamic excitation tests on the GO networks, the specific test procedure of which is listed in SI. As shown in Figure S13, the GO networks maintained good integrity after being excited by a 50 nm amplitude sweep frequency at indentation depths of 3 and 10 µm. Meanwhile, we also extracted the loss factor, storage modulus, and loss modulus of the GO networks during the excitation process, as shown in Figure S14. It can be seen that the loss factor of GO networks gradually increases with the increase of the excitation frequency, reaching 0.01 near 40 Hz. Since melamine foam contains many such energy dissipation pathways, when melamine foam is subjected to vibration excitation, the skeletal deformation effectively activates these energy dissipation pathways, resulting in an effective enhancement of the damping properties. Moreover, as presented in Figure 4d (3 mg/mL sample), foam skeleton with adhered GO exhibited intrinsic wrinkled features, while siloxane species attached to the surface of GO further increased surface roughness. The increased roughness effectively enhanced frictional energy dissipation during interfacial slippage. Figure 4e illustrates a schematic diagram of the damping enhancement mechanism.

Along with dynamic energy dissipation features, energy absorption capabilities of various melamine foam samples under quasi‐static compression were also explored, which was measured by the area under the stress‐strain curves within 0–40% strain range. As shown in Figure S15, the energy absorption values of the pure, 2, 3, and 4 mg/mL samples were 0.34, 0.52, 1.40, and 1.16 MPa, respectively. These results indicated that, with the introduction of GO networks, foam samples were able to absorb greater amounts of deformation energy under the same compressive strain. The significantly enhanced energy absorption capacity suggested considerable potential for practical applications such as impact protection, cushioning, and vibration mitigation.

### 2.4 Imparting Waterproof Properties to Melamine Foam

Melamine foam inherently exhibits excellent flame‐retardant properties; however, it suffers from poor water resistance. Water absorption can lead to issues such as mold growth, which severely limits its practical applications. Interestingly, after assembling a silane coupling agent‐assisted inter‐skeletal GO network, melamine foam exhibits significant hydrophobicity. As shown in Figure 5a, when a water droplet was deposited onto the surface of the pure sample using a syringe, the droplet was rapidly absorbed by the foam (Video SI). In contrast, as shown in Figure 5b, when a water droplet was placed on the surface of a sample containing GO networks, the droplet remained stable on the surface (Video SII). For quantitative evaluation of the wettability of surface, contact angles of GO network‐integrated samples with diiodomethane and water were measured, as presented in Figure 5e‐j. The results indicate that the sample exhibits selective wetting behavior, rather than general liquid repulsions. The increased apparent water contact angle of the GO network‐integrated melamine foam suggests suppressed water penetration; while the contact angle of diiodomethane is significantly lower than that of water, indicating a stronger affinity for non‐polar liquids. Figure 5c shows the obtained statistical contact angle results. For samples with higher GO concentrations, contact angles with both water and diiodomethane were slightly lower than those of samples with lower GO concentrations. The calculated surface energies of different samples are presented in Figure 5d, where the 3 mg/mL sample exhibited the highest surface energy.

**FIGURE 5:**
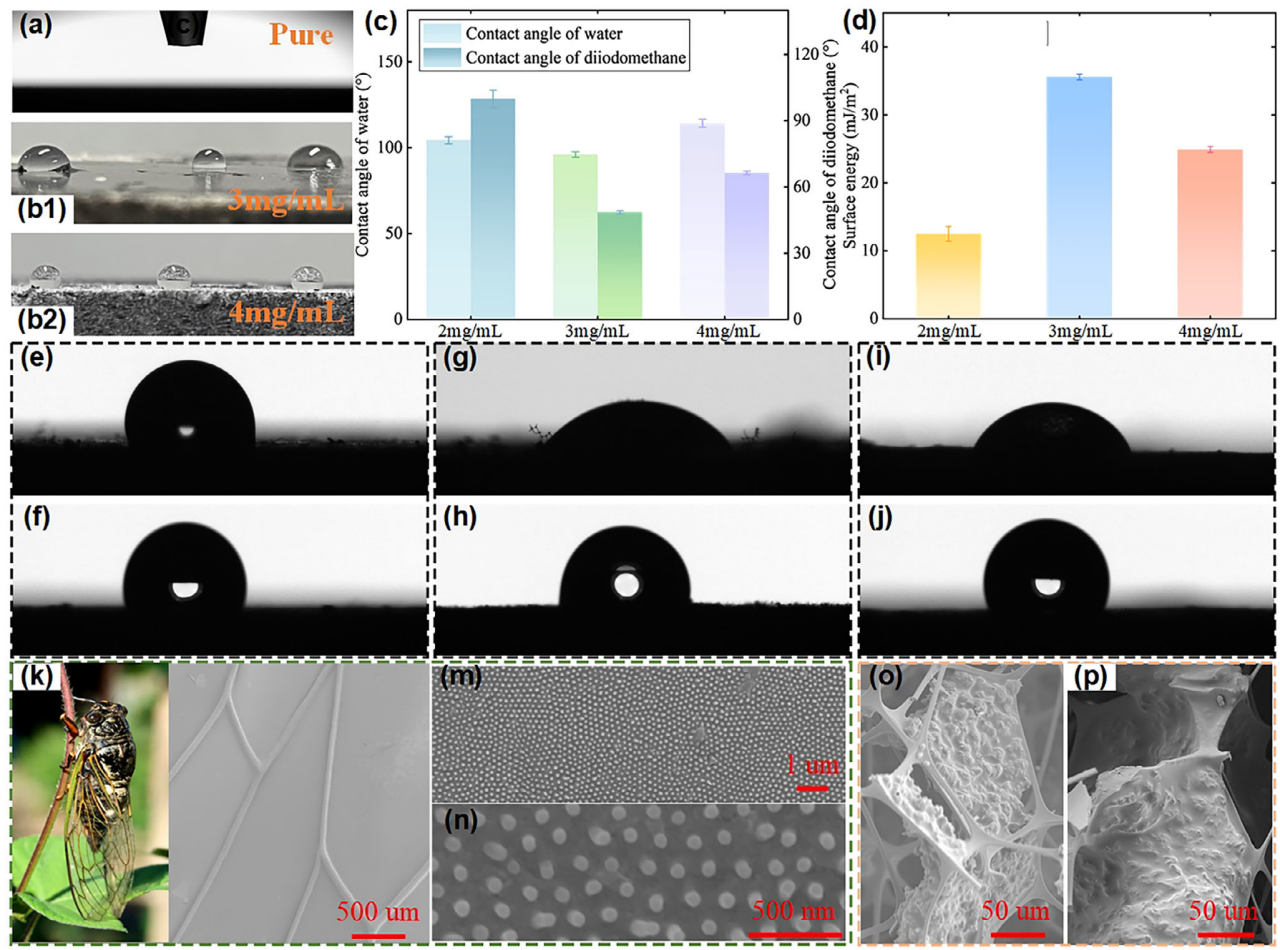
Waterproof properties and surface characteristics of melamine foam with GO networks. Optical microscopy image of a water droplet on the a) Pure sample and b) GO network‐integrated sample. c) Contact angles of different samples with water and diiodomethane (Data in panels c and d are presented as mean ± SD, with n = 3 independent specimens for each group. Error bars represent SD). d) Comparison of surface energies for different samples. Optical microscopy images showing the contact angles with diiodomethane and water of the e), f) 2 mg/mL sample; g), h) 3 mg/mL; i), and j) 4 mg/mL sample, respectively. k), m) and n) SEM images of cicada wings. o), and p) SEM images of the 3 mg/mL sample, illustrating the surface micro‐protrusions.

To elucidate the origin of this wetting behavior, we conducted additional control experiments using modified foams with only GO (named GO‐only) and only silane coupling agents (named KH550‐only). As shown in Figures S16 and S17, for the GO‐only sample, deionized water was rapidly absorbed upon contact with the sample surface, indicating that unreduced GO itself cannot impart hydrophobicity to melamine foam. This is because GO contains abundant oxygen‐containing groups, enabling it to interact with water through hydrogen bonds and polar interactions. In contrast, as shown in Figures S18, S19, and S22a, the KH550‐only sample exhibited an apparent water contact angle as high as 134.37°. The organ siloxane structure formed after the hydrolysis and condensation of silane coupling agents can cover the GO surface, reducing direct contact between hydrophilic sites and water, thus forming a hydrophobic interface layer. Besides the chemical effect of KH550, the microstructural features of the GO network between the frameworks also contribute to the hydrophobicity. As shown in Figure 5k–n, micro protrusions exist on the cicada wings, which are believed to contribute to its hydrophobicity. Similarly, as shown in Figure 5o, p (3 mg/mL sample), the wrinkles and localized stacking of GO sheets on the surface of the GO network‐integrated melamine foam create micro‐protrusions and depressions. When a water droplet contacts this rough surface, it cannot completely fill all the micro‐grooves and depressions. Instead, the water droplet is partially supported by the protrusions, while air is trapped in the depressions, forming a Cassie‐Baxter type solid‐air‐water composite interface. Due to the relatively high surface tension of water, penetrating these narrow depressions requires overcoming capillary pressure barriers. Therefore, the trapped air pockets reduce the effective solid‐liquid contact area, inhibiting water penetration into the porous foam and resulting in an increased apparent water contact angle.

As shown in Figure S20, for the GO‐only sample, diiodomethane is rapidly absorbed, behaving similarly to deionized water. For the KH550‐only sample, as shown in Figures S21 and S22b, the contact angle of diiodomethane is 90.88°, significantly lower than its contact angle with deionized water. These results indicate that the silane coupling agent‐derived organ siloxane interface can effectively reduce water wettability, but does not impart the same repulsiveness to nonpolar liquids with low surface tension. Diiodomethane can still wet the surface because it interacts with the siloxane structure and rough surface regions through dispersion interactions.

In summary, the wettability of GO network‐integrated melamine foams should be understood as selective wetting. The organ siloxane interface derived from the silane coupling agent primarily provides hydrophobicity, while the GO network introduces wrinkles, rough surfaces, carbon‐rich regions, and residual oxygen‐containing groups. Therefore, the foam exhibits significant hydrophobicity but can still be wetted by diiodomethane.

### 2.5 Effective Enhancement of Sound Absorption Performance

Melamine foam is also widely used as a sound absorption material. Its sound absorption performance is shown in Figure 6a, exhibiting a typical open‐cell foam behavior, where the sound absorption coefficient was increased by increasing frequency. It was evident that the in situ formed GO networks structures across the melamine foam skeletons could effectively enhance its sound absorption performance. For instance, at 5000 Hz, the sound absorption coefficient of the pure sample was 0.3997, whereas that of the 3 mg/mL sample reached 0.745. To measure sound absorption performance enhancement, noise reduction coefficient (NRC) was adopted as a comprehensive metric, which was defined as the average sound absorption coefficient at 250, 500, 1000, and 2000 Hz. Figure 6b shows the derived NRC values, where the NRC value of the 2 mg/mL sample was 0.1745, representing an improvement of about 31.7% compared with that of the pure sample (0.1325). Furthermore, sound absorption performance was significantly enhanced with the increase of melamine foam thickness, as presented in Figure 6c,d. When sample thickness exceeded 30 mm, the 2 mg/mL sample presented sound absorption coefficients of above 0.9 over 1500 to 6000 Hz broad frequency. Figure 6e illustrates NRC variation with sample thickness, presenting an almost linear relationship between thickness and NRC. The mechanisms by which GO networks enhanced melamine foam sound absorption performance is schematically illustrated in Figure 6g. For the pure sample, incident sound wave‐induced air vibrations could pass through the foam structure in a relatively straight‐through manner, limiting energy dissipation. However, for GO network‐integrated samples, two primary mechanisms increased sound energy dissipation. First, the presence of GO networks partially blocked airflow channels, resulting in more tortuous airflow paths and significantly higher flow resistance, increasing viscous dissipation. Notably, SEM revealed that GO networks did not completely block melamine foam skeleton; instead, a number of pores remained within the networks (Figure 6h). This configuration was equivalent to the in situ construction of numerous microscale, distributed, and highly damped Helmholtz resonator arrays within the foam. These resonant units dissipated sound energy through air column oscillations under sound excitation, effectively suppressing sound wave propagation. Moreover, the coexistence of networks and foam skeletons with different characteristic dimensions contributed to broadband sound absorption. Second, GO networks was not perfectly smooth and many GO “fins” protruded from the networks surface, as shown in Figure 6f, which further increased flow resistance. Meanwhile, sound excitation can induce microscale vibrations of the GO networks, efficiently converting sound energy into strain energy within the networks and frictional heat at interfaces, thereby achieving effective noise reduction. Moreover, At 2 mg/mL, the GO networks spans across the foam skeleton, but due to the low GO concentration, it does not excessively seal the open‐cell structure of the melamine foam itself. This condition is more conducive to the penetration of low‐to‐mid‐frequency sound waves through the porous network. Simultaneously, the coupled vibration of the foam skeleton and the GO networks, as well as interfacial friction and viscous dissipation, jointly promote sound wave dissipation. Conversely, at 3 mg/mL, the GO networks becomes more continuous, leading to increased airflow resistance and enhanced viscous dissipation, which is more beneficial for high‐frequency sound absorption. Therefore, the difference in GO networks morphology due to different GO concentrations further affects the sound wave energy dissipation mechanism and causes the structure's sound absorption characteristics to exhibit differences across different frequency bands. The high‐density coating of GO networks may also partially block the pores or even increase reflection, which explains why the sound absorption performance does not increase monotonically with concentration across the entire frequency range.

**FIGURE 6:**
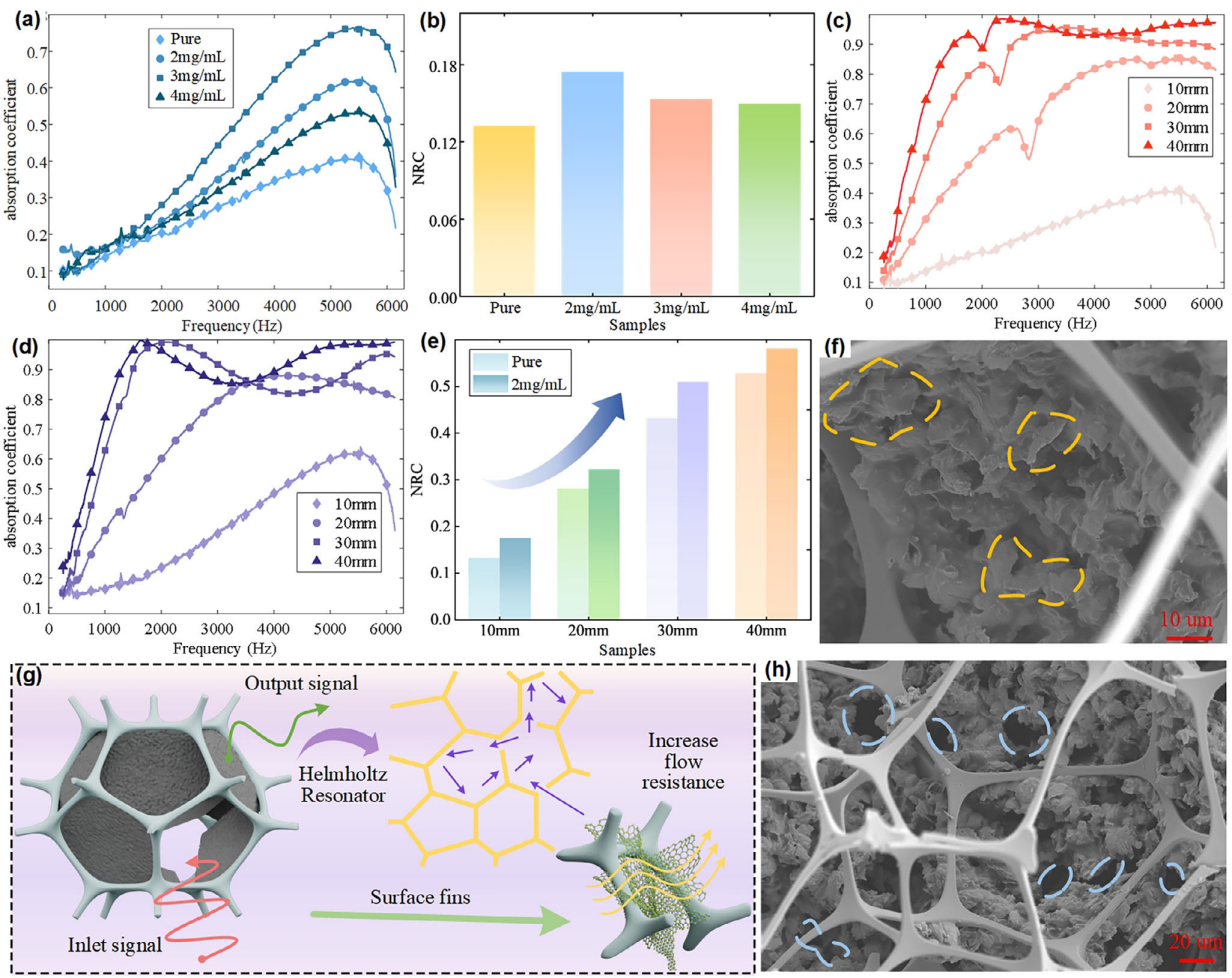
Sound absorption performance and enhancement mechanisms of melamine foam with GO networks. a) Sound absorption curves of different samples with a thickness of 10 mm. b) NRC values calculated from the corresponding sound absorption curves in panel a. Sound absorption performance of c) Pure sample and d) 2 mg/mL sample at different thicknesses. e) NRC values calculated from the corresponding sound absorption curves in panels c and d. f), and h) SEM images of the 3 mg/mL sample. g) Schematic illustration of the mechanism underlying the GO network‐induced sound absorption enhancement.

## 3 Conclusion

In summary, inspired by cicada wing skeleton‐networks architecture, this research presented a new approach to enhance melamine foam properties. By in situ assembling inter‐skeleton GO networks within the foam, the damping, mechanical, and sound absorption characteristics of the foam were simultaneously enhanced. After modification, foam compressive modulus was increased by about 924%, allowing it to sustain loads up to 1000 times its weight without considerable deformation; vibration attenuation time was decreased by about 83.3% compared to the unmodified sample, facilitating rapid transition from a vibrational state to a stable state. In addition, GO networks effectively enhanced the sound absorption properties of the foam. Interestingly, the originally water‐absorbent melamine foam became hydrophobic after modification, showing effectiveness against both nonpolar and polar liquids. This research provided a new approach for foam structure multifunctional enhancement and demonstrated distinct advantages in applications requiring simultaneous improvements in waterproofing, damping, mechanical, and sound absorption properties.

## 4 Experimental Section

### 4.1 Sample Preparation

GO networks were prepared using a dip‐coating process based on the process schematically shown in Figure S23. The raw material used in this preparation process was commercially available GO, as shown in Figure S24, the GO sheet size used in this study is approximately 5‐10 µm. During the process, the mixture solution was mixed through mechanical stirring at a constant speed for 5 min to promote the KH‐550 hydrolysis and the dispersion of GO, where silane groups underwent hydrolysis in an aqueous environment, followed by Si‐O‐Si condensation generating localized siloxane networks. Meanwhile, the ‐NH_2_ groups at one end of the silane coupling agent reacted with oxygen‐containing groups on GO surface through a ring‐opening reaction, producing stable C‐N covalent bonds. These interactions, combined with π–π stacking interactions among GO sheets as well as hydrogen bonding between GO and silane coupling agent, promoted the assembly of GO sheets into a network‐like structure. In addition, the hydrolyzed silanol groups ‐Si(OH)_3_ could undergo condensation reactions with ‐NH‐ groups on melamine foam skeleton surface, producing Si‐O‐N and Si‐O‐C bonds. This process effectively anchored GO networks onto the melamine foam framework, fabricating GO‐network‐incorporated melamine foam. In this research, a series of samples were fabricated by changing GO dispersion concentration and the prepared samples were labelled based on their GO concentration as Pure, 2, 3, and 4 mg/mL samples. Figure S25 shows optical images of the different samples. As could be clearly seen, the overall color of the samples became progressively darker by increasing the concentration of GO.

A detailed experimental characterization section can be found in the Supporting Information.

## Author Contributions

**Jigang Feng**: conceptualization, methodology, validation, writing – original draft, funding acquisition. **Anhao Li**: methodology, validation. **Babak Safaei**:  writing – review and editing, investigation, funding acquisition. **Yun Kong**: Writing – review and editing, validation. **Yizhen Miao**: validation. **Zhaoye Qin**: conceptualization, methodology, project administration, writing – review and editing, resources, supervision. **Fulei Chu**: writing – review and editing, supervision.

## Funding

China Postdoctoral Science Foundation (Grant No.2025M781306); European Union under the REFRESH‐Research Excellence For REgion Sustainability and High‐tech Industries project number CZ.10.03.01/00/22_003/0000048 via the Operational Programme Just Transition.

## Conflicts of Interest

The authors declare no conflict of interest.

## Supporting information




**Supporting File 1**: advs76720‐sup‐0001‐SuppMat.docx.

**Supporting File 2**: advs76720‐sup‐0002‐VideoS1‐S2.zip.

**Supporting File 3**: advs76720‐sup‐0003‐SoundS1‐S2.zip.

## Data Availability

The data that support the findings of this study are available from the corresponding author upon reasonable request.
