# Supplementary material for: Cicada Wing‐Inspired Skeleton‐GO Networks in Melamine Foams for Multifunctional High‐Performance Composites
Source: Adv Sci (Weinh). 2026 Jul 24;13(57):e76720. doi: 10.1002/advs.76720 (PMC13397819; doi:10.1002/advs.76720)
Supplement: Supplementary file 1 [file ADVS-13-e76720-s002.docx]

Supporting Information

**Cicada Wing-Inspired Skeleton- GO Networks in Melamine Foams for Multifunctional High-Performance Composites**

Jigang Feng^1^, Anhao Li^1^, Babak Safaei^2, 3^, Yun kong^4^, Yizhen Miao^1^, Zhaoye Qin^1*^, Fulei Chu^1^

^[1]1^ State Key Laboratory of Tribology, Department of Mechanical Engineering, Tsinghua University, Beijing, China

^2^ Department of Mechanical Engineering, Eastern Mediterranean University, Famagusta, North Cyprus via Mersin 10, Türkiye

^3^ Department of Machining, Assembly and Engineering Metrology, Faculty of Mechanical Engineering, VSB-Technical University of Ostrava, 70800, Ostrava, Czech Republic

^4^ School of Mechanical Engineering, Beijing Institute of Technology, Beijing, 100081, China

* Corresponding author email: [qinzy@mail.tsinghua.edu.cn](mailto:qinzy@mail.tsinghua.edu.cn)

ORCID: 0000-0003-3892-4594.

**Fabrication routes:**

Melamine foam was cut into samples with different shapes and dimensions. Rectangular specimens with 30 mm × 30 mm × 15 mm dimensions were fabricated for compression tests and those with 13 mm × 13 mm × 5 mm dimensions were prepared for dynamic mechanical analysis (DMA). For sound absorption measurements, cylindrical samples 100 and 29 mm in diameters with 10, 20, 30, and 40 mm thicknesses were prepared. The same specimens used for compression tests were employed for waterproof performance evaluation, while vibration tests were conducted using the samples prepared for sound absorption measurements. Silane coupling agent (KH-550, 3-aminopropyltriethoxysilane) and 95% ethanol were obtained from Shanghai Macklin Biochemical Co., Ltd. All other materials were applied with no further purification. Graphene Oxide was obtained from Nanjing JCNANO Tech Co., Ltd.

As shown in **Figures S1**, a mixed ethanol-water solution containing 12 wt% silane coupling agent was first prepared. GO was then added to the solution at 2, 3, and 4 mg/mL concentrations. The mixture was then mixed through stirring at a constant speed for 5 min to promote the KH-550 hydrolysis and the initial dispersion of graphene. Therefore, the prepared melamine foam specimens were placed in the solution and further stirred gently for 20 min. Then, specimens were dried in an oven at 50 ℃ to facilitate ethanol and water evaporation.

During stirring process, the oxygen-containing groups on GO, likely including epoxy groups, react with the amino groups of KH-550, thereby forming C-N related interfacial bonds.

C-O-C + -NH_2_ → C-N + OH

This reaction, along with π-π stacking interactions among GO sheets and hydrogen bonding interactions between KH-550 and GO, collectively promoted continuous graphene network formation.

Furthermore, the hydrolyzed silanol groups likely underwent condensation reactions on melamine foam skeleton surface, strengthening interfacial bonding between foam framework and graphene network.

Si-OH + Surface-OH/NH →Si-O-Surface + H_2_O

These reactions collectively facilitated stable graphene network adhesion onto melamine foam, incorporating graphene networks with melamine foam.

**Characterization：**

The morphologies of graphene networks and melamine foam were evaluated using scanning electron microscopy (SEM, ZEISS GEMINISEM 300) and elemental distribution was characterized using energy-dispersive spectroscopy (EDS) equipped on the SEM. Chemical bonding characteristics of various samples were analyzed using X -ray photoelectron spectroscopy (XPS, Thermo Fisher ESCALAB 250Xi). Thermal stability of the samples was explored using Thermogravimetric analysis (TGA, TA Instruments Q5000IR) over a temperature range from room temperature to 600°C, with a heating rate of 10°C/min. Acoustic absorption was measured using an impedance tube system (Shanghai Swat Acoustic Technology Co., Ltd.) according to a standardized two-microphone random excitation procedure and ASTM E1050, and using two impedance tubes of different diameters for measurements in different frequency ranges. Compressive mechanical properties were tested using an Instron E3000 universal testing machine. Dynamic mechanical behaviors were characterized by a Waters Q800 dynamic mechanical analyzer (DMA) using a three-point bending fixture to determine sample frequency responses over the range of 1-85 Hz.

**Figure S1.** Surface morphology of 3 mg/mL sample.

**Figure S2**. SEM images of 4mg/mL.

**Figure S3.** SEM images of a)2mg/mL, b)3mg/mL, c) and d)4mg/mL.

The TGA test were conducted in an air atmosphere, the temperature was increased from room temperature to 500 ℃ at a rate of 10 ℃/min.

**Figure S4.** TGA curves of a)various samples and b)GO.

**Figure S5.** FTIR curves of various samples.

**Figure S6.** a) Comparison of the XPS spectra of different samples; b) High-resolution C1s XPS spectrum of GO.

**Figure S7.** XPS analysis of different samples: a) C1s peak of Pure sample; b) N1s peak of Pure sample; c) C1s peak of 4 mg/mL sample; d) N1s peak of 4 mg/mL sample.

**Figure S8.** High-resolution Si2p XPS spectrum of the 4 mg/mL sample.

**Figure S9.** Storage modulus varies with frequency.

As shown in **Figure S10**a, in-situ compression of the GO network across melamine foam were performed using a nanoindenter. Specifically, the nanoindenter was Keysight Technologies G200, with a circular indenter end face and a diameter of 9.08 μm, the cross-sectional shape of which is shown in **Figure S10**b.

**Figure S10**. a) Schematic illustration of the in-situ nanoindentation compression of the GO network; b) schematic of the nanoindenter tip.

**Figure S11.** Comparison of optical microscopy images before and after compression of 4mg/mL at a compression depth of 10 μm.

**Figure S12.** Force-displacement curves of the GO network during compression: a) and b) 4mg/mL.

In-situ dynamic excitation experiments were also conducted. Specifically, after the indenter of the indenter tip contacted the surface of the GO network, it continued to indent, and two compression depth are set, 3 μm and 10 μm, respectively, wiht an indentation speed of 50 nm/s. After indenting to the specified distance, the nanoindentation was subjected to a sinusoidal frequency sweep with an amplitude of 50 nm and a frequency of 1-45 Hz, with 10 sweep points.

**Figure S13.** Comparison of optical microscopy images before and after dynamic excitation of 4mg/mL at different compression depths: a) compression depth of 3 μm; b) compression depth of 10 μm.

**Figure S14.** Dynamic mechanical responses of the 4mg/mL at different compression depths: a) loss factor, b1) storage modulus, and b2) loss modulus at a compression depth of 3 μm; c) loss factor, d1) storage modulus, and d2) loss modulus at a compression depth of 10 μm.

**Figure S15.** Energy absorption capability of different samples (n = 3 independent specimens for each group. Error bars represent SD).

**Figure S16.** SEM of GO-only.

**Figure S17**. Photographs of water on the GO-only sample at different contact times.

**Figure S18**. SEM of KH550-only.

**Figure S19**. Water contact angles on the KH550-only sample at three different positions.

**Figure S20.** Photographs of diiodomethane on the GO-only sample at different contact times.

**Figure S21**. Diiodomethane contact angles on the KH550-only sample at three different positions.

**Figure S22**. Contact angles of the KH550-only sample with a) water and b) diiodomethane.

**Figure S23.** Schematic illustration of the preparation of melamine foam with graphene networks.

**Figure S24**. SEM images of GO

**Figure S25.** Optical images of different samples.

**References**

[1]	Y. Wang, Z. Chen, Y. Lu, et al., "Apparent activation energy and characteristic temperatures of thermal decomposition research of microwave prepared melamine foam," *J Polym Res*, 30 (2023): 336, https://doi.org/10.1007/s10965-023-03714-6.

[2]	C. Yan, Y.-J. Luo, W.-G. Zhang, et al., "Preparation of a novel melamine foam structure and properties," *Journal of Applied Polymer Science*, 139 (2022): 51992, https://doi.org/10.1002/app.51992.
